# Performance Evaluation of a High-Precision Low-Dose Powder Feeder

**DOI:** 10.1208/s12249-020-01835-5

**Published:** 2020-11-03

**Authors:** Sara Fathollahi, Stephan Sacher, M. Sebastian Escotet-Espinoza, James DiNunzio, Johannes G. Khinast

**Affiliations:** 1 Research Center Pharmaceutical Engineering (RCPE) GmbH, 8010 Graz, Austria; 2 Institute of Process and Particle Engineering, Graz University of Technology, 8010 Graz, Austria; 3 Oral Formulation Sciences and Technology, Merck & Co., Inc., Rahway, New Jersey USA

**Keywords:** continuous feeding, low-dose feeding, low-dose API, free flowing/cohesive powder

## Abstract

**Abstract:**

Highly potent active pharmaceutical ingredients (APIs) and low-dose excipients, or excipients with very low density, are notoriously hard to feed with currently available commercial technology. The micro-feeder system presented in this work is capable of feeding low-dose rates of powders with different particle sizes and flow properties. Two different grades of lactose, di-calcium phosphate, croscarmellose sodium, silicon dioxide, a spray-dried intermediate, and an active ingredient were studied to vary material properties to test performance of the system. The current micro-feeder system is a volumetric feeder combined with a weighing balance at the outlet that measures feeder output rates. Feeding results are shown as a so-called “displacement-feed factor” curve for each material. Since the powder mass and volume are known in the micro-feeder system, in this work, we characterized an observed density variation during processing *via* a “displacement-feed factor” profile for each of the fed powders. This curve can be later used for calibrating the system to ensure an accurate, constant feed rate and in addition predicting feeding performance for that material at any feed rate. There is a relation between powder properties and feeding performance. Powders with finer particles and higher compressibility show densification during their feeding process. However, powders with larger particles and lower compressibility show both “densification” and “powder bed expansion,” which is the manifestation of dilation and elastic recovery of particles during the micro-feeding process. Through the application of the displacement-feed factor, it is possible to provide precise feeding accuracy of low-dose materials.

**Graphical abstract:**

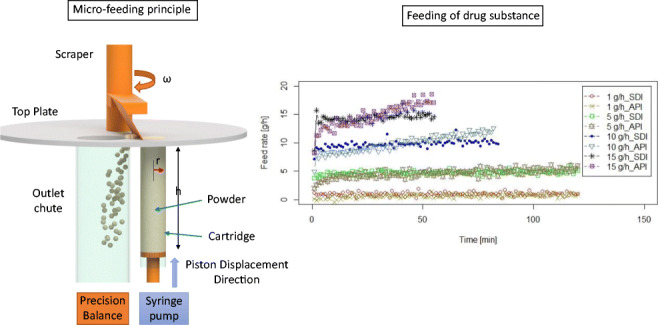

## INTRODUCTION

Continuous feeding of small quantities of powder is a challenge in a wide range of processes and industries, including the pharmaceutical industry (1,2). Especially, in the context of continuous manufacturing, continuous feeding of materials is one of most critical unit operations in the entire line. While continuous feeding of fluids is a fairly easy challenge, creating a constant powder stream is challenging, especially for sticky and cohesive materials. Advantages of continuous manufacturing in terms of reduced footprint, advanced quality, and scalability are well known (3,4). In the last years, several modular continuous manufacturing implementations have been commercialized by different equipment companies, including GEA, Glatt, or Bosch to name a few. In addition, personalized, individualized, and small-scale manufacturing is increasingly important, also in the context of translational pharmaceutics.

Common to all continuous manufacturing operations is the need to create continuous powder flows (5–8). While feed rates of kilograms per hour can be attained using standard equipment, low feed rates in the range of grams per hour are difficult to achieve due to the intermittent nature of granular flows from small-scale screw conveyors. Even more problematic is the continuous feeding of cohesive or electrostatic materials, which flow in chunks or agglomerates. However, continuous low-dose feeding is increasingly important for two reasons: first, the pharmaceutical industry is in the process of adopting continuous manufacturing. Second, high-potency active pharmaceutical ingredients (HPAPIs), with doses in the range of milligrams or even micrograms per tablet, are becoming more frequent (9–11). Currently, no reliable equipment exists that can be used for continuous low-dose feeding of powders (12–16). Continuous feeding of fine powder at the low ratios needed for a given formulation is one of the key issues in continuous manufacturing processes (10,17).

Depending on the specific material density, fine powders with a particle size below 50–100 μm experience significant cohesive forces between individual particles (17). Van der Waals forces, electrostatic forces, and capillary forces are predominant in the dispersion of fine powders (17). These forces can lead to agglomeration and adhesion of particles to the walls of feeders (17). However, many APIs are powders with particles in the cohesive range. Thus, especially the continuous feeding of APIs in a continuous manufacturing framework is challenging, if not impossible with current systems. An overview of available micro-feeders and their feeding principle is provided in a previous study (18).

Powders with larger particles and better flow properties are relatively easy to feed by conventional loss-in-weight (LIW) feeders with feed rates in the range of 0.5–100 kg/h (10). However, the precision of such processes must be carefully monitored and feed rate deviations from the set point must be minimized by optimizing the process parameters (10,19). In addition to feed rate fluctuations that occur due to the fact that powder flows are always to a certain degree discrete, feed rate deviations happen in the LIW feeders due to refilling of the feeders (19,20). During refilling, LIW feeders operate in “blind-flight” volumetric mode, although efforts are made by feeder companies to allow for a “smart refill”, which essentially is a prediction of the feed rate during refilling based on past experience. Of course, the accuracy of a volumetric feeding mode is strongly influenced by the variability of powder packing density (1). Density of powders is a strong function of powder properties, such as particle size distribution, shape, packing properties, of processing history, as well as environmental conditions such as vibration and humidity (1,21). Even batch-to-batch variations in the powder properties may introduce errors in the feeding system.

In this study, we introduce a micro-feeding system based on the volumetric principle combined with external feed rate monitoring by a high-precision balance. This system enables feeding powders with different particle sizes and powder properties for feed rates from below 1 g/h to about 100 g/h. The micro-feeder system contains a cartridge with a moveable piston inside. The feed rate is controlled *via* the displacement of the piston inside the cartridge. Since the cartridge dimensions and powder mass are known in this system, the feed rate can be adjusted by changing the displacement rate. Moreover, the total amount of powder fed over time is constant and known. The presented work is a follow-up from the data presented by Besenhard et al. (22). A more advanced design of the micro-feeder system with a number of improvements compared with the previous one was developed (22). This includes the miniaturization of the system, control of the displacement rate *via* a controlled precision syringe pump, and automated data recording. This work presents a detailed analysis of feeding performance of the novel micro-feeder in relation to the fed material properties.

Specifically, the feeding performance for two different grades of lactose, di-calcium phosphate, croscarmellose sodium, silicon dioxide, a model API, and a spray-dried intermediate (SDI) was tested. The first three powders are common excipients and silicon dioxide (a flow aid) is reported to be difficult to feed due to its low density and strong electrostatic behavior (10). The API is low density crystalline material with poor flow characteristics. The SDI was selected for its cohesive and low bulk density characteristics which is representative of other SDI materials. Using these materials, this study aimed to characterize and assess the performance of the micro-feeder system.

## MATERIALS AND METHODS

### Materials

Two α-lactose-monohydrate excipients (CapsuLac60 and GranuLac200 from Meggle, Germany), di-calcium phosphate (dibasic calcium phosphate, Sigma-Aldrich, UK), croscarmellose sodium (sodium carboxymethylcellulose, Sigma-Aldrich, UK), silicon dioxide (CAB-O-SIL M-5P fumed silica, Cabot Switzerland GmbH, Switzerland), one active pharmaceutical ingredient, API A, and one spray-dried intermediate, SDI B (both provided by Merck & Co., Inc., Kenilworth, NJ, USA, known as MSD outside of the USA and Canada) were used in this study.

### Micro-feeder System

**Fig. 1:**
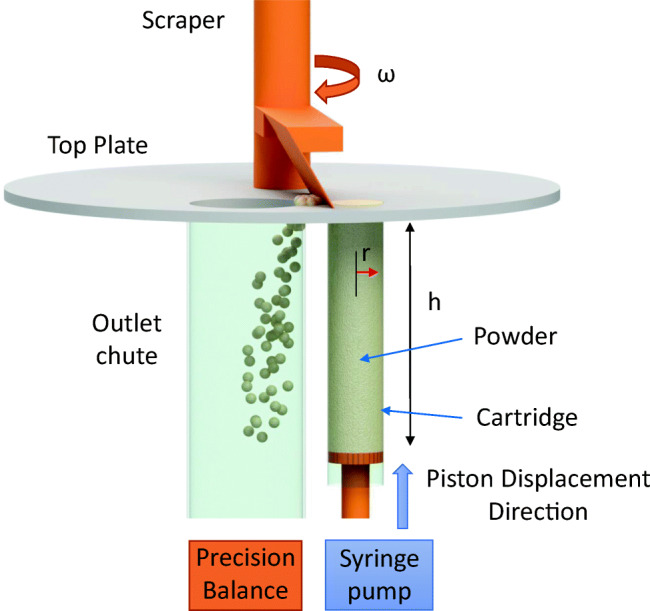
Principle of the micro-feeder system

The micro-feeder system presented in this study is based on advanced “volumetric” feeding principle, as shown in Fig. 1. The micro-feeder contains a highly polished metal cartridge (using polishing “paper 420”), into which the powder is filled, with a moveable Teflon piston, a linear actuator, and a scraper. Speed of the piston in the cartridge can be exactly controlled by the linear actuator *via* a syringe pump. Displacement of the piston in the cartridge replaces the powder from the cartridge in an exact way (volumetric filling principle).

Since the total amount of powder in the cartridge, the cross-sectional area of the tube and the piston displacement speed are known, the volumetric displacement rate over the process is constantly monitored and, in conjunction with a density value, can be used to calculate the mass flow rate. However, because density variations of the packed material occur, the instantaneous mass flow rate varies accordingly. Density variations can occur due to two effects: first, uneven distribution of the powder during filling and preparation of the cartridge. And second, due to a local densification during feeding caused by wall friction. Thus, the critical success factor for using such a device in routine manufacturing is to design the filling procedure in a way that density variations along the cartridge axis are minimized. Moreover, wall friction should be kept low in order to reduce “in-process densification”. Alternatively, weight loss over time can be monitored (*i.e.*, “loss-in-weight principle”) to control the piston speed. This, however, can be avoided with appropriate design of the micro-feeder.

As shown in Fig. 1, in addition to the motor-controlled piston and the cartridge, the system consists of a top plate with two openings. The first opening is for the cartridge powder to exit the cartridge. The second one is a hole for the powder to fall into a chute from which it is fed to the process. A scraper pushes powder from the first opening into the hole. Thus, periodically, exiting powder is transferred to the feeding chute. An anti-static kit integrated in the system can be used to prevent scattering or sticking of the powder material during the transfer process onto the balance.

In the present study, a cartridge with the following dimensions was used: *r* = 11.5 mm, *h* = 100 mm. Clearly, longer/shorter and wider cartridges may be used, depending on the intended filling task.

In summary, this novel feeding system relies on four main principles:

- Volume at the bottom is continuously and constantly displaced
- Initial density in the cartridges is as constant as possible
- In-process densification is kept to a minimum by reducing wall friction
- The average feed rate is always known each second and the actual feed rate will not surpass a certain percentage threshold, which is guaranteed by the first three arguments

### Experimental Setup and Detailed Analysis of Feeding Process

In our experimental setup, powder exits the cartridge on the top (due to piston displacement) and is removed by a scraper by pushing the powder into an opening in the top plate. By gravity, it falls through a hole into the chute and then further on a precise balance (Mettler Toledo, XPE204, 0–220 g). For all experiments shown in this work, the accumulated powder mass was recorded every second automatically using “BalanceLink” software (Mettler Toledo, version 4.1.3). Feed rates were determined once per second and converted to g/h from the generated data (${\overset{.}{m}}_{\mathrm{f}}=\varDelta m/\varDelta t$). Due to the periodic scraper action, powder falling through the chute onto the balance fluctuates; the average feed rate per one (1) minute was used for further analysis.

Displacement of the piston at the bottom of the cartridge within 1 min (*ΔV*) and the accumulation of mass at the scale (relating to powder exiting at the top) within 1 min (*Δm*) are known. Volumetric displacement at the top of the cartridge is not known precisely, although it will be similar (yet not necessarily identical) to *ΔV*. We thus define a property called “effective displacement density” as $\frac{\varDelta m}{\varDelta V\ }$, which is a good approximation of the actual bulk density of the powder at the exit*.* We normalized it by the tapped density *ρ*_T_ and called it “relative effective displacement density”, ${\rho}_{\mathrm{ED}}^{\ast }$. Thus,

1 $$ {\rho}_{\mathrm{ED}}^{\ast }=\frac{\varDelta m}{\varDelta V\ {\rho}_{\mathrm{T}}} $$

### Micro-feeding Process

In order to try and maintain a constant mass flow rate, the volumetric displacement feeding unit requires a pre-conditioning of material to reduce the powder density variation along the cartridge’s axis. The poured bulk density of materials (*ρ*_B_) is a function of the filling procedure, powder particle packing property, and wall friction effect. It also changes during manipulation and thus will change during feeding since particles will rearrange, leading to a densification during feeding. Without pre-conditioning, four process phases exist in the presented micro-feeder:

1. Starting phase: a lag time is observed due to compaction of the powder by piston displacement and wall friction. The duration depends on the bulk density after the filling process and the powder state.
2. Increasing feed rate: the feed rate increases until the powder is compacted to a “saturated volume” in the cartridge due to the frictional effects.
3. Stable feed rate: the feed rate stays constant. This period can be used for steady process feeding.
4. Process end: the feed rate drops, when the powder in the cartridge is depleted.

In order to avoid densification during the feeding process (*i.e.*, steps 1 and 2), a pre-conditioning procedure was developed. During pre-conditioning, the powder is densified to the tapped density to achieve saturated density faster.

### Pre-conditioning

The pre-conditioning procedure consists of the following steps: after pouring powder in the cartridge, finger tapping (strokes by the side) and refilling of powder are performed as long as densification can be observed. This step helps powder particles to pack more effectively. Subsequently, by closing the upper/open part of the cartridge by a plate (lid) and raising the piston for a defined distance (*h*_0_, see Eq. 2), the powder is compacted until the tapped density is achieved. Since powder mass in the cartridge (*m*) and cartridge volume (*V*_c_) are known, the tapped density of the material (value is known from powder characterization, see below) is achieved by compression. The piston displacement for reducing the volume of the powder mass in the cartridge (the defined distance of *h*_0_) is calculated as:

2 $$ {h}_0=\frac{\left(M-m\right)\ h}{M} $$

where *h* is the cartridge height (in this study *h* = 100 mm) and *M* is the mass of powder that could be filled in the cartridge at tapped density (*M* = *V*_c_ *ρ*_T_). After removing the lid on top and before starting the micro-feeding process, the powder that spills out on the top is removed and recorded. This is a small amount only that can be neglected for further analysis.

During the feeding process, the piston displacement in the cartridge pushes the powder upwards. The feed rate is controlled by the piston speed. The piston speed set point is calculated based on the amount of powder in the cartridge after pre-conditioning and the desired feed rate. The piston’s displacement rate (mm/min) is controlled by the syringe pump, leading to a constant volumetric displacement.

The micro-feeder device was tested using powders with different particle sizes and properties to assess the range of industrial applicability. Feeding performance, reproducibility, and impact of various critical process parameters (*e.g.*, syringe pump speed and scraper angular speed) were studied. After pre-conditioning, feeding consistency was tested for three fixed feed rate set points of 5, 10, and 15 g/h. Each feed rate set point was adjusted by a fixed piston displacement speed, which was calculated from the initial density in the cartridge and the desired feed rate. For all materials, the lowest possible displacement speed of 0.1 mm/min was tested. Due to the low density of silicon dioxide, feed rates of 1.5 and 2.5 g/h—calculated from the density and volumetric displacement—were studied. All experiments were performed in triplicate.

The scraper pushes the powder periodically to the vertical chute. The scraper speed defines the time interval of powder falling on the balance. For all experiments done in this study, the scraper speed was set to 10 rpm (which equals one rotation every 6 s) and the material amount discharged to the “process” is recorded in a 1-s interval. Constant set-up and parameters (except displacement) were used for all feeding experiments. Pre-conditioning (finger tapping and pre-compaction to achieve the material tapped density) was done for all test runs.

### Material Characterization

#### Particle Size Distributions

HELOS/KR (OASIS/L dry dispersing system Sympatec, Clausthal-Zellerfeld, Germany) was used to measure the particle size distributions of materials. HELOS is a laser light diffraction technique that is capable of measuring particle size range of 0.45 to 875 μm. All measurements were done in triplicate.

#### Powder Density Measurement

Pharmatest PT-TD200, a standardized method described in the United States Pharmacopeia (USP 2011, h616i), was used to measure the bulk (poured) and tapped density of materials. The bulk density (*ρ*_B_, g/cm^3^) was measured by pouring powder in a standard 250-mL cylinder. After mechanically tapping the powder, the tapped density was determined (*ρ*_T_, g/cm^3^). Hausner ratio (***H***_***R***_ ***=*** *ρ*_T_/*ρ*_B_), the ratio of the tapped to the bulk density, is a common parameter, to indicate the flowability of powders (23). All measurements were done in triplicate to determine powder density.

#### FT4 Powder Rheometer

A FT4 powder rheometer system (Freeman Technology, Tewkesbury, UK) was used to measure the wall friction angle at 15 kPa stress. The standard wall friction test procedure was applied using the 25-mm diameter FT4 glass vessel. A stainless steel disk with a roughness of 0.28 μm was used for this measurement, in order to represent the highly polished stainless steel cartridge of the micro-feeder.

Furthermore, the FT4 powder rheometer system was used in this study to measure the elastic behavior of materials. The elastic behavior is measured by recording the powder bed height while undergoing direct uniaxial, vertical compression of 15 kPa, followed by unloading. This compression method is mentioned in the literature as “quasi-static testing” method (24). In this test, the bed height was recorded at the beginning (powder bed height at zero-pressure, *L*_0_), starting from both the poured and tapped state. Then, a direct pressure of 15 kPa was applied to the powder bed and the height was recorded (powder bed height at-pressure, *L*_1_). The difference between the powder bed height at zero pressure and at the applied pressure (in percentage) is the compressibility of powder at 15 kPa. After removing the load, the powder bed height was recorded once again (*L*_2_). These data provide useful information on the elastic behavior of the powder bed.

Powder bed height data of the materials were measured starting from poured powder state and tapped state. ΔL % at 15 kPa pressure is also termed compressibility at 15 kPa. This is a common measurement for compressibility and is reported in many studies (25–28). However, it is not reported how the powder bed expands after removing the pressure (*i.e.*, compressibility after removing the 15 kPa pressure). The “difference between compressibility at-pressure and after removing it” is defined as the elastic recovery of powders. This value defines the powder bed height expansion (in percentage) after removing the direct pressure, *i.e.*, the elastic recovery.

## RESULTS AND DISCUSSION

Materials with different particle sizes and properties were used to represent a wide range of powder properties. For example, CapsuLac60 (*d*_50_ = 244 μm) and GranuLac200 (*d*_50_ = 31 μm), both being α-lactose-monohydrate excipients, were used to represent opposite material behavior in terms of flowability (free flowing *vs.* cohesive). GranuLac200 with a ***H***_**R**_ greater than 1.22 (23) exhibits poor flowability. In contrast, CapsuLac60 has good flowability (***H***_**R**_ = 1.10). Lactose is a common filler for tableting and capsule filling in pharmaceutical industry. Di-calcium phosphate (*d*_50_ = 184 μm) and croscarmellose sodium (*d*_50_ = 43 μm) are common excipients in pharmaceutical formulations for tableting. Silicon dioxide (*d*_50_ = 20 μm) is a multifunctional excipient for pharmaceutical solid dose manufacturing. It is commonly used as an anti-caking and anti-blocking agent, spray aid, carrier, thickening, and stabilization agent.

**Fig. 2:**
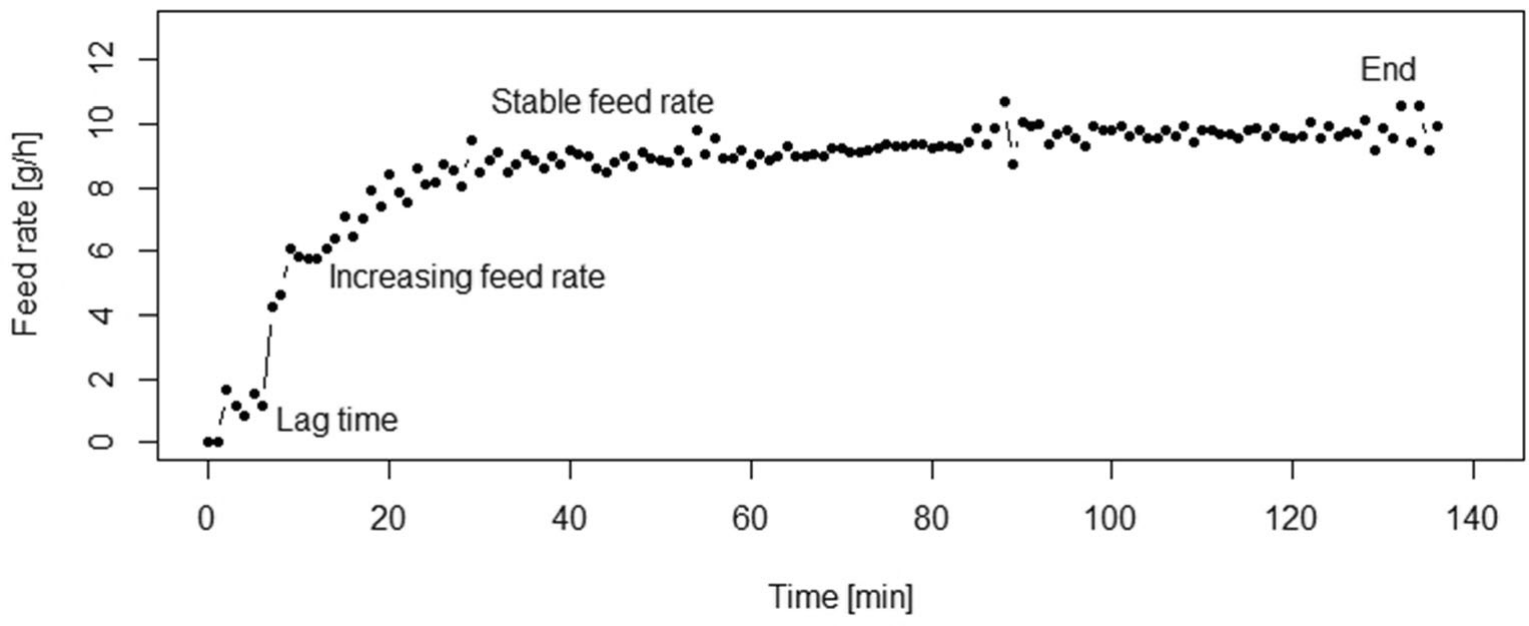
Feeding process without pre-conditioning step. Material: di-calcium phosphate; feed rate of 10 g/h (displacement speed: 0.46 mm/min)

In Fig. 2, all four process phases (described above) can be seen during feeding of di-calcium phosphate without pre-conditioning (*i.e.*, filled close to the poured density *ρ*_B_). For all materials in this section, pre-conditioning was done prior to feeding process. The detailed information on pre-conditioning is summarized in Table I.

**Table I:** Detailed Pre-conditioning Information for All Used Materials. ± Represents One Standard Deviation (*n* = 3)

Materials	CapsuLac60	Di-calcium phosphate	Croscarmellose sodium	GranuLac200	Silicon dioxide
Bulk (poured) density ***ρ***_B_ [g/cm^3^]	0.68 ± 0.00	0.70 ± 0.00	0.51 ± 0.00	0.52 ± 0.00	0.04 ± 0.00
Tapped density ***ρ***_T_ [g/cm^3^]	0.75 ± 0.00	0.87 ± 0.00	0.77 ± 0.00	0.93 ± 0.00	0.05 ± 0.00
Powder density after finger tapping and refilling [g/cm^3^]	0.67 ± 0.01	0.82 ± 0.01	0.69 ± 0.01	0.55 ± 0.01	0.04 ± 0.00
Powder in the cartridge [g]^*a*^	27.85 ± 0.63	33.90 ± 0.42	28.56 ± 0.35	26.69 ± 0.40	1.55 ± 0.04
*h*_0_ [mm]^*b*^	10.61 ± 2.01	6.47 ± 1.17	10.23 ± 1.09	41.27 ± 1.04	23.94 ± 2.08
Powder spill after pre-conditioning [g]	0.18 ± 0.06 (0.6%)	0.22 ± 0.07 (0.6%)	0.45 ± 0.23 (1.6%)	0.26 ± 0.04 (1%)	0.03 ± 0.03 (1.9%)
Powder density in the cartridge before starting the feeding process [g/cm^3^]	0.75 ± 0.00	0.87 ± 0.00	0.73 ± 0.00^c^	0.93 ± 0.00	0.05 ± 0.00

^*a*^After finger tapping and refilling

^*b*^Calculated from Equation 2

^*c*^For croscarmellose, only a density of 0.73 g/cm^3^ could be achieved in the cartridge

**Fig. 3:**
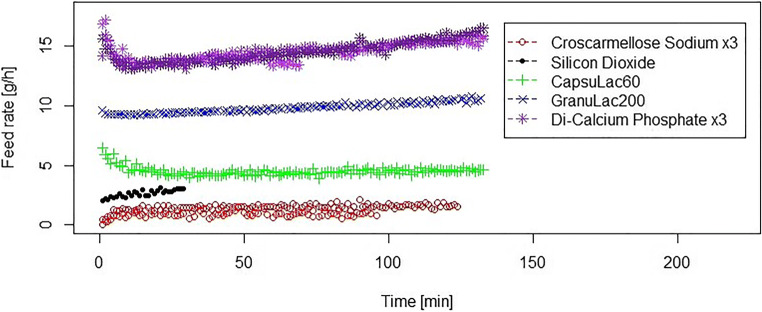
Overview of feeding experiments in the micro-feeder. Feed rate of 2.5 g/h for silicon dioxide, 5 g/h for CapsuLac60, and 10 g/h for GranuLac200 are shown. All triplicate test runs (TR) are shown for croscarmellose sodium (feed rate of 1.5 g/h) and di-calcium phosphate (feed rate of 15 g/h). Short feeding duration for silicon dioxide is due to very low bulk density of this powder

Figure 3 shows an overview of the feeding performance for the five different materials. For better visibility, reproducibility (defined as the repeatability of the feeding curves for a particular displacement condition) is only shown for the lowest and highest feed rates in Fig. 3. Note that the duration of feeding of silicon dioxide is shorter than other materials due to the very low density of silicon dioxide (0.04 g/cm^3^). Powder properties of materials (before feeding process) are summarized in Table II.

**Table II:** Powder Properties of Investigated Materials (Before Feeding Process). ± Represents 1 Standard Deviation (*n* = 3)

Materials	CapsuLac60	Di-calcium phosphate	Croscarmellose sodium	GranuLac200	Silicon dioxide
X10 [μm]	121.5 ± 5	14.6 ± 1	19.5 ± 0	4.7 ± 0	7.6 ± 0
X50 [μm]	243.7 ± 6	184.4 ± 5	43.4 ± 0	30.5 ± 0	20.2 ± 0
X90 [μm]	426.6 ± 30	314.5 ± 3	119.5 ± 2	102.2 ± 0	75.2 ± 1
Hausner ratio (***H***_**R**_)	1.10	1.24	1.51	1.79	1.25
Wall friction angle [°]	25.28 ± 0.83	25.58 ± 0.17	28.79 ± 0.94	28.90 ± 0.44	^a^

^*a*^SiO_2_ showed difficulties during FT4 rheometer system (Freeman Technology, Malvern, UK) measurements

From Fig. 3, different conclusions can be drawn: first, feeding performance is highly reproducible for a given powder. Second, feed rates between 1.5 and 15 g/h can be obtained. Third, for a constant piston speed, the feed rate deviations do not exceed 10–20% for the total course of feeding. Lastly, the characteristics (shape) of the feed rate *versus* time curve are sensitive to powder properties. Di-calcium phosphate and CapsuLac60 (similar properties, see Table II and Table III) have higher feed rates at the beginning, followed by a decrease, and later, an increase, suggesting an initial expansion of the powder bed, followed by densification. However, for GranuLac200 and croscarmellose sodium, feed rate increases continuously, suggesting a continuous densification during feeding. Silicon dioxide behaves similarly.

**Table III:** A Summary of the Powder Bed Height Data Starting from Powder Poured State (Parameters Were Used to Define the Elastic Behavior of Materials). Experiments Were Done in Triplicate

Materials	*L*_0_ [mm]	*L*_1_ [mm]	*L*_2_ [mm]	Compressibility at 15 kPa pressure (*L*_0_-*L*_1_)/*L*_0_ [%]	Compressibility after removing the pressure (*L*_0_-*L*_2_)/*L*_0_ [%]	Difference between at pressure and after removing the pressure^a^ [%]
CapsuLac60	19.03 ± 0.03	18.30 ± 0.10	18.53 ± 0.09	4	3	32
Di-calcium phosphate	19.03 ± 0.01	18.34 ± 0.06	18.56 ± 0.06	4	2	32
GranuLac200	19.08 ± 0.02	11.89 ± 0.20	12.03 ± 0.20	38	37	2
Croscarmellose sodium	19.06 ± 0.03	17.26 ± 0.08	17.56 ± 0.07	9	8	17

*L*_*0*_ powder bed height at zero pressure, *L*_*1*_ powder bed height at 15 kPa pressure, *L*_*2*_ powder bed height after removing the pressure

^*a*^Elastic recovery of powder bed [(*L*_2_-*L*_1_)/(*L*_0_-*L*_1_)]

Powder bed height data of the materials are summarized in Table III and Table IV, respectively, starting from the poured powder state and tapped state. As can be seen, CapsuLac60 and di-calcium phosphate show some compressibility for the poured initial state. GranuLac200 had significant compressibility and croscarmellose sodium had a compressibility of 9%. Elastic recovery was moderate in all cases. This is interesting since the materials with high compressibility greatly densified upon pressure without significant elastic recovery, *i.e.*, internal structure was modified and internal voids were irreversibly destroyed due to rearrangement and shear. For the tapped density states, small compressibility was observed, with some elastic recovery which in relative terms was between 40 and 50%. For silicon dioxide, FT4 measurements could not be performed (no data are available for this powder in both Table III and Table IV).

**Table IV:** A Summary of the Powder Bed Height Data Starting from Powder Tapped State (Parameters Were Used to Define the Elastic Behavior of Materials). Experiments Were Done in Triplicate

Materials	*L*_0_ [mm]	*L*_1_ [mm]	*L*_2_ [mm]	Compressibility at 15 kPa pressure (*L*_0_-*L*_1_)/*L*_0_ [%]	Compressibility after removing the pressure (*L*_0_-*L*_2_)/*L*_0_ [%]	Difference between at pressure and after removing the pressure^a^ [%]
CapsuLac60	19.50 ± 0.06	19.08 ± 0.06	19.28 ± 0.05	2	1	49
Di-calcium phosphate	19.78 ± 0.19	19.21 ± 0.10	19.47 ± 0.15	3	2	46
GranuLac200	19.68 ± 0.16	19.11 ± 0.07	19.33 ± 0.05	3	2	40
Croscarmellose sodium	19.95 ± 0.08	19.27 ± 0.09	19.56 ± 0.05	3	2	43

*L*_*0*:_ powder bed height at zero pressure, *L*_*1*_ powder bed height at 15 kPa pressure, *L*_*2*_ powder bed height after removing the pressure

^*a*^Elastic recovery of powder bed [(*L*_2_-*L*_1_)/(*L*_0_-*L*_1_)]

### Detailed Analysis of Feeding Experiments

**Fig. 4:**
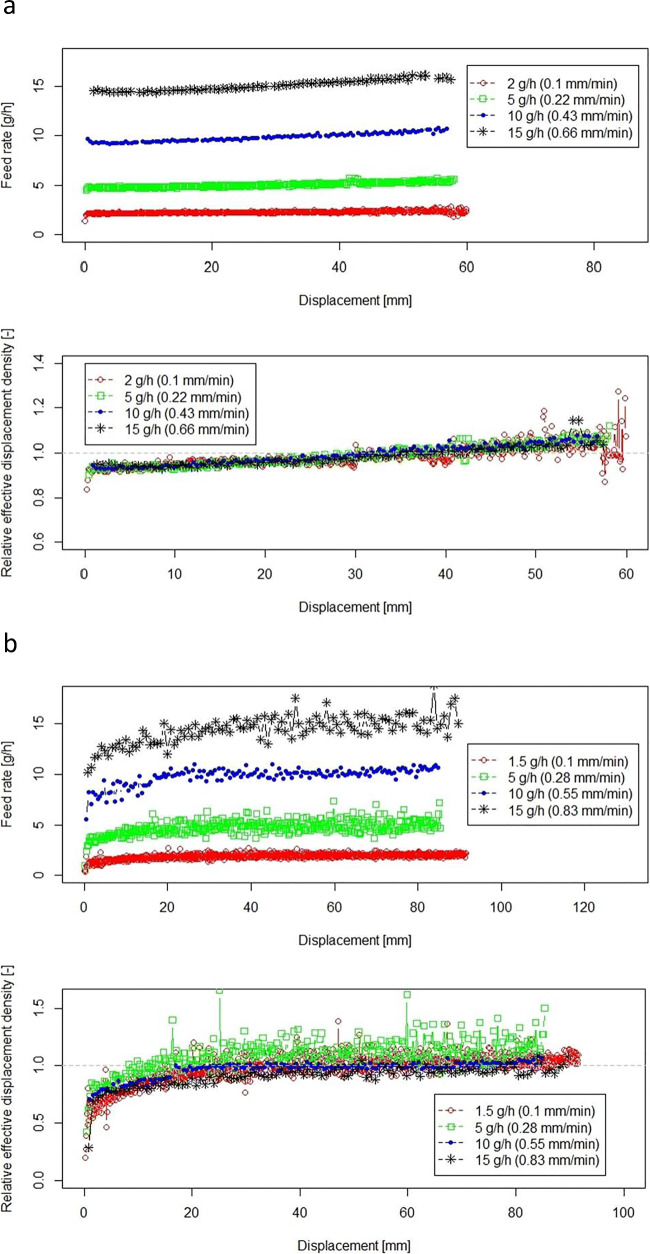
Feeding of GranuLac200 (**a**) and croscarmellose sodium (**b**) at different feed rates. Feed rate (upper plot) and relative effective displacement density (lower plot) during the feeding process *versus* displacement. The “relative effective displacement density” data are shifted to origins (*h*_0_, pre-conditioning compaction range of 41.27 ± 1.04 mm for GranuLac200 and 10.23 ± 1.09 mm for croscarmellose sodium). Displacement speed is given in brackets for each feed rate

In this section, the feeding performance of powders with different powder properties is discussed. Feed rates and “relative effective displacement density, ${\rho}_{\mathrm{ED}}^{\ast }$,” for both GranuLac200 and croscarmellose sodium are shown in Fig. 4 a and b, respectively, for various feed rates. Different displacement speeds (given in brackets) were used to obtain the feed rate. For a better comparison, the feed rate and effective displaced density are plotted *versus* displacement instead of time, since feed rates with slower displacement speed of 0.1 mm/min take a much longer time.

For both fine powders, *i.e.*, GranuLac200 and croscarmellose sodium, the feed rate increases slightly over the feeding process. There are two explanations: possibly, a density gradient was created during pre-conditioning or powder densification occurs along the cartridge during feeding (due to the friction at the walls). Likely, both effects contribute to this observation.

As can be seen in Fig. 4 a and b, the first material leaving the cartridge was below tapped density (${\rho}_{\mathrm{ED}}^{\ast }$ < 1), and during feeding, the density increased to a value greater than tapped density (${\rho}_{\mathrm{ED}}^{\ast }$ > 1). Note that the average ${\rho}_{\mathrm{ED}}^{\ast }$ needs to be 1, since the cartridge was compressed to obtain powder of exactly tapped density. As can be seen in Fig. 4 a and b, the feed rates initially increase significantly.

For both GranuLac200 and croscarmellose sodium, the feed rate slope (top plots) seems steeper for higher feed rates. However, the ${\rho}_{\mathrm{ED}}^{\ast }$ curves (bottom plots) show the same increase in slope for different feed rates. This plot thus enables a better understanding of the process. The feed rate is equal to ${\rho}_{\mathrm{ED}}^{\ast }$ times the displaced volume per time, which is a constant. The plots show that ${\rho}_{\mathrm{ED}}^{\ast }$ for both GranuLac200 and croscarmellose sodium (see Fig. 4 a and b) evolve independently of the feed rate (displacement speed). According to Amonton’s law of friction, the frictional forces are directly proportional to the normal forces, independently of the area of contact and the sliding velocity (29). Thus, the results agree with this law. Most importantly, every powder has the same densification profile, which once measured can be used to control the piston speed. In summary, feed rate changes are mild at constant piston speed.

**Fig. 5:**
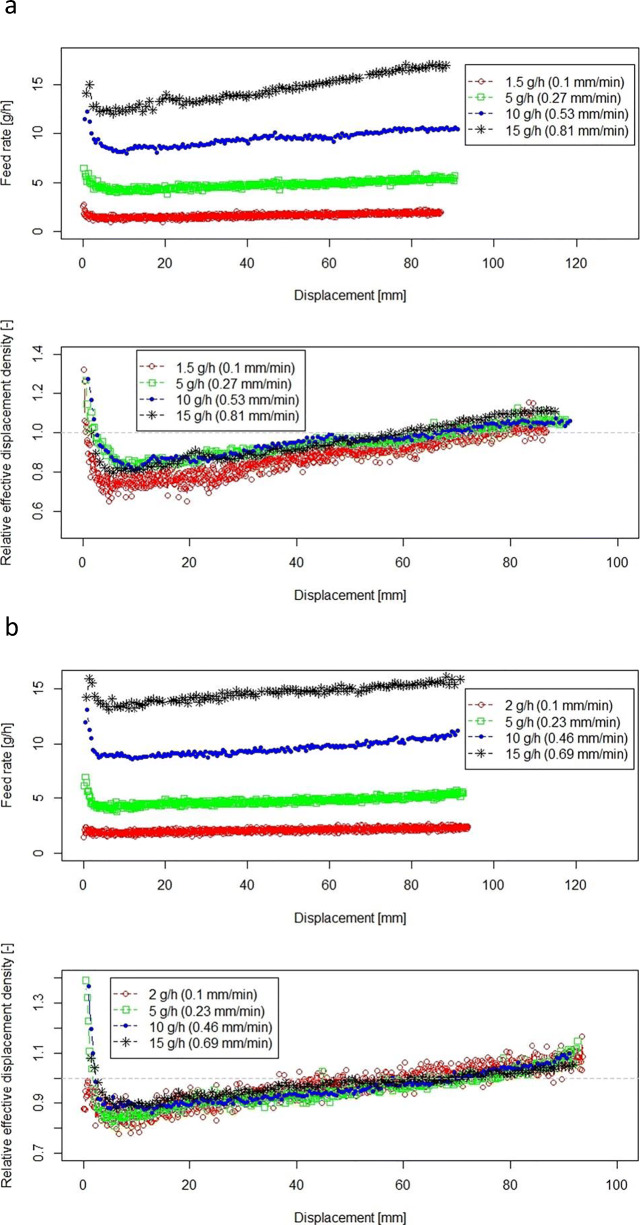
Feeding of CapsuLac60 (**a**) and di-calcium phosphate (**b**) at different feed rates. Feed rate (upper plot) and relative effective displacement density (lower plot) during feeding process *versus* displacement. The relative effective displacement density data are shifted to origins (*h*_0_, pre-conditioning compaction range of 10.61 ± 2.01 mm for CapsuLac60 and 6.47 ± 1.17 mm for di-calcium phosphate). Displacement speed is given in brackets for each feed rate

Feeding process and relative effective displacement density, ${\rho}_{\mathrm{ED}}^{\ast },$ for CapsuLac60 and di-calcium phosphate are shown in Fig. 5 a and b, respectively. In contrast to the other finer powders, the feed rate for both powders decreases at the beginning to a minimum level and then it increases again. Feeding performance of CapsuLac60 and di-calcium phosphate is thus significantly different from GranuLac200 and croscarmellose sodium. CapsuLac60 and di-calcium phosphate have larger particles, lower compressibility, and larger elastic recovery compared with GranuLac200 and croscarmellose sodium (see powder properties in Table II and Table III).

As can be seen from Table II and Table III, the powder bed height of CapsuLac60 decreased 4% at 15 kPa direct pressure (see Table III). However, after removing the pressure, the particle bed expanded and the powder bed height increased by 32%. In contrast, GranuLac200’s elastic recovery was low, at only 2% powder bed expansion. Croscarmellose sodium’s elastic recovery was about 17%, and thus, also much lower than the recovery of CapsuLac60 and Di-calcium phosphate (32%). For the compression analysis from the tapped state, the trends were similar, yet not as pronounced. Thus, we hypothesize that the observed initial high feed rate and high density are due to a non-uniform expansion of the powder bed in the cartridge after the pre-conditioning. This effect led to displacement of more mass per time interval at the beginning of the feeding process, manifesting as initially higher feed rates, followed by a lower density/feed rate phase.

Slight differences in the effective displacement density curves may be a result of small differences of powder mass in the cartridge (see Table I). This led to slightly different density levels in the cartridge. The similar shape of the curves supports this hypothesis.

**Fig. 6:**
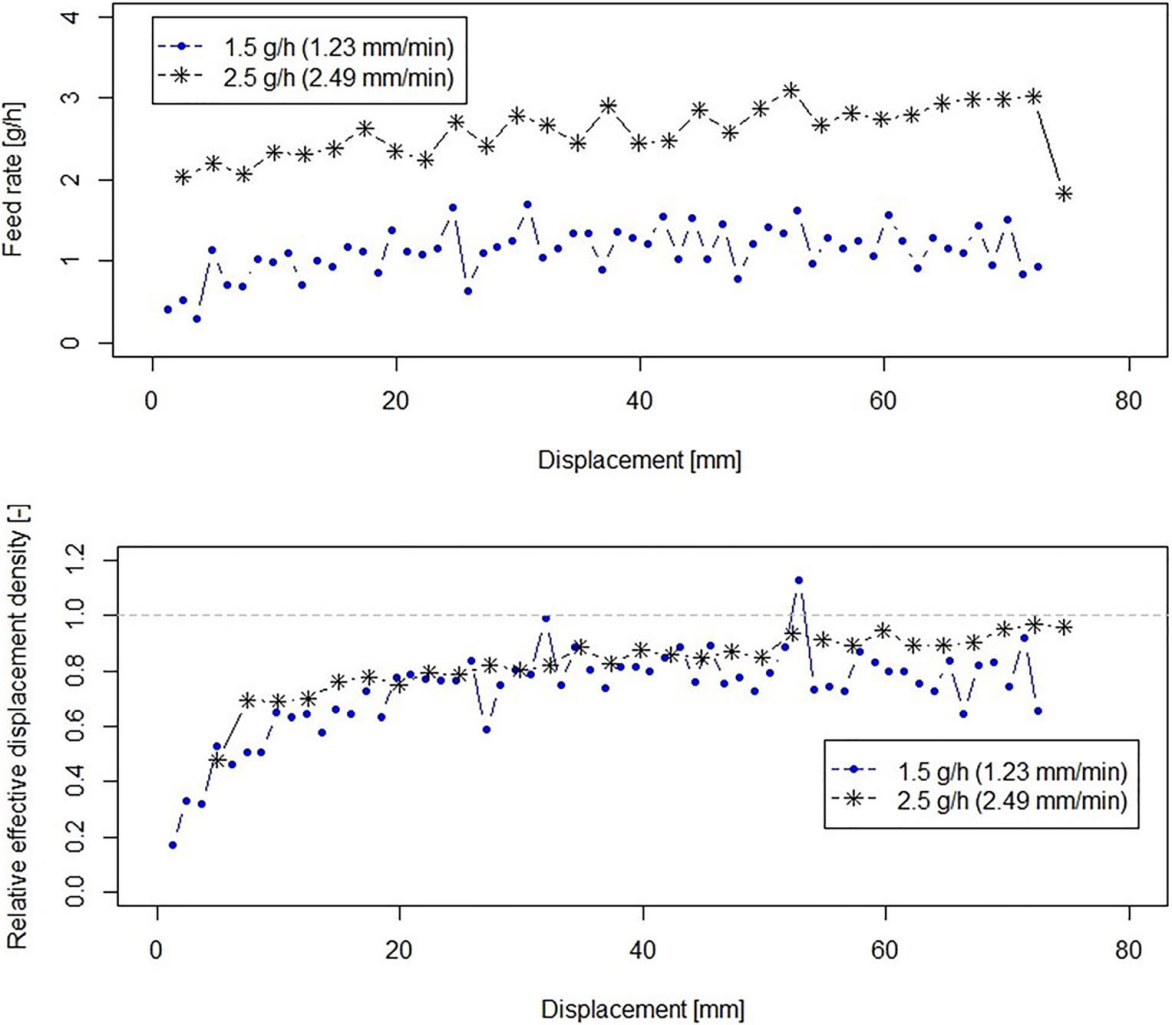
Feeding of silicon dioxide at different feed rates. Displacement speeds are given in brackets for each feed rate. Feed rate (upper plot) and relative effective displacement density (lower plot) during feeding process *versus* displacement. The relative effective displacement density data are shifted to origins (*h*_0_, pre-conditioning compaction range of 23.94 ± 2.08 mm)

The feeding experiments for silicon dioxide are shown in Fig. 6. Silicon dioxide is reported to be difficult to handle due to its low density, high cohesion, and electrostatic properties (10). However, the novel micro-feeder was capable of feeding the glidant at feed rates of 1.5 and 2.5 g/h without significant fluctuations where similar behavior was noted to the other fine powders tested in this study. As can be seen in Fig. 6, the relative effective displacement density, ${\rho}_{\mathrm{ED}}^{\ast }$, of silicon dioxide is slightly less than unity (1). Due to very low bulk density (0.04 g/cm^3^) and electrostatic properties, some material may have been lost during experimental execution. Alternatively, the resolution of the scale might have been insufficient for the low weights associated with silicon dioxide.

Generally, it was found that powders with larger particles and lower compressibility show first a higher feed rate, and therefore, initial densification during the micro-feeding process. In contrast, powders with finer particles and higher compressibility only slightly densify during the feeding process. Thus, feeding performance of powders is dependent on the particle size distributions, bulk density, compressibility, and elastic behavior of powders. However, the plots of the relative effective displacement density, ${\rho}_{\mathrm{ED}}^{\ast },$ over displacement are similar for all different feed rates for all investigated materials. Thus, once this curve has been measured for one powder for one feeding rate, displacement speed can be controlled such that a uniform feed rate can be obtained. Using the ${\rho}_{\mathrm{ED}}^{\ast },$ curve as a “displacement-feed factor calibration” for programming the syringe pump can ensure a constant feed rate. Average of the relative effective displacement density, ${\rho}_{\mathrm{ED}}^{\ast }$, and relative standard deviation (RSD) for all investigated materials are summarized in Table V.

**Table V:** Summary of ${\rho}_{\mathrm{ED}}^{\ast }$ [−] ± RSD [%] for All Investigated Material at Discrete Points of 25, 50, and 75 mm Displacement

Materials/displacement	CapsuLac60	Di-calcium phosphate	Croscarmellose sodium	GranuLac200	Silicon dioxide
25 mm	0.82 ± 6%	0.90 ± 3%	1.03 ± 17%	0.97 ± 1%	0.81 ± 4%
50 mm	0.93 ± 3%	0.96 ± 2%	1.05 ± 9%	1.05 ± 5%	0.82 ± 4%
75 mm	1.02 ± 3%	1.02 ± 2%	1.05 ± 9%	^a^	^a^

^*a*^No data is available at this discrete point (see *h*_0_ range in captions of Figs. 4 and 6)

### Segregation in the Micro-feeder System

Due to the filling procedure, pre-conditioning, and feeding, powder segregation may occur. CapsuLac60 has a wide particle size distribution and is relatively free-flowing which may increase the likelihood of size segregation. In order to study the segregation along the cartridge in the micro-feeder system, samples of 3 g were taken during the feeding process. First sample (3 g) was taken at the beginning, then the next sample (3 g) in the middle, and the last one (3 g) at the end of the feeding process. The particle size distributions of these samples were measured subsequently by QICPIC (OASIS/L dry dispersing system Sympatec, Clausthal-Zellerfeld, Germany). Table VI shows the particle size distributions of the CapsuLac60 samples taken at different times during the feeding process for two different feed rates of 20 and 75 g/h. No significant difference in particle size distribution was noted between the collected data and the data summarized in Table II (before feeding process). Since no size segregation for CapsuLac60 (having the highest segregation potential) was observed, we assume that all other materials also do not exhibit segregation.

**Table VI:** Particle Size Distribution of CapsuLac60 Samples Taken at the Beginning, in the Middle and at the End of the Feeding Process for Two Different Feed Rates of 20 and 75 g/h

Material	Feed rate [g/h]	Sample taken	X10 [μm]	X50 [μm]	X90 [μm]
CapsuLac60	20	At the beginning	143	242	355
		In the middle	140	240	353
		At the End	141	240	351
CapsuLac60	75	At the beginning	137	235	347
		In the middle	137	239	350
		At the End	133	234	344

### Micro-feeding of Industrial API and SDI

**Fig. 7:**
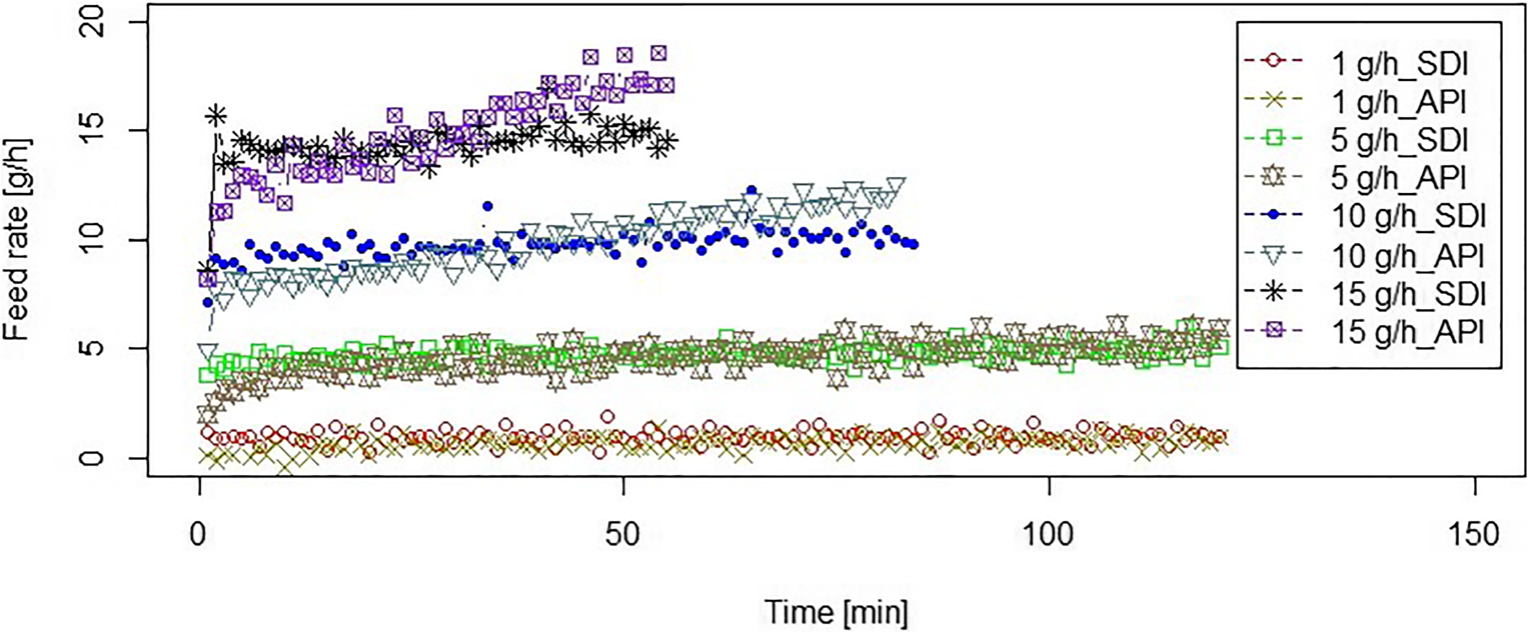
Micro-feeding of commercial APIs. The displacement speeds for API to adjust the feed rates of 1, 5, 10, and 15 g/h are, respectively, 0.1, 0.5, 0.99, and 1.49 mm/min**.** The displacement speeds for SDI are, respectively, 0.1, 0.46, 0.93, and 1.39 mm/min for adjusting 1, 5, 10, and 15 g/h feed rates

API A and SDI B were used as received to expand the feasibility study for industrial relevant API and SDI, respectively. The particle size distribution and detailed pre-conditioning information of these materials are summarized in Table VII. Based on the PSD and ***H***_**R**_, we assume that both materials are highly cohesive and sticky (30). Figure 7 shows the low-throughput feeding performance for both API and SDI (following the micro-feeding process section) for a maximum feeding duration of 2 h. For feed rates of 10 and 15 g/h, the feeding duration is shorter due to the available powder mass in the cartridge.

**Table VII:** Particle Size Distribution and Detailed Pre-conditioning Information of the Active Ingredients (API and SDI) Provided by Merck & Co., Inc. (MSD)

Materials	API A (API)	SDI B (SDI)
X10 [μm]	8.13	21
X50 [μm]	19.11	42
X90 [μm]	38.65	79
Bulk (poured) density ***ρ***_B_ [g/cm^3^]	0.25 ± 0.00	0.32 ± 0.00
Tapped density ***ρ***_T_ [g/cm^3^]	0.41 ± 0.00	0.44 ± 0.00
Hausner ratio (***H***_**R**_)	1.64	1.38
Powder density after finger tapping and refilling [g/cm^3^]	0.34 ± 0.00	0.34 ± 0.00
Powder in the cartridge [g]^a^	13.94 ± 0.12	14.02 ± 0.15
*h*_0_ [mm]^b^	17.13 ± 0.70	22.77 ± 0.82
Powder spill after pre-conditioning [g]	0.03 ± 0.00 (0.2%)	0.12 ± 0.02 (0.9%)
Powder density in the cartridge before starting the feeding process [g/cm^3^]	0.40 ± 0.00	0.43 ± 0.00

^*a*^After finger tapping and refilling

^*b*^Calculated from Equation 2

Again, performance was reproducible for both API and SDI over the 3 runs performed at each displacement rate. SDI B had a stable constant feed rate at lower flow rates, but deviations were noted for the 10 and 15 g/h set points. Similarly, API A showed deviations for higher feed rates of 10 and 15 g/h. This feeding behavior is similar to the GranuLac200 (see Fig. 4a) and croscarmellose sodium (see Fig. 4b) feeding. The possible reasons are explained above in “Detailed analysis of feeding experiments” section.

With these results, we have shown that the potential industrial applicability of the micro-feeder system was evaluated using fine and cohesive APIs at various low feed rates of 1, 5, 10, and 15 g/h. It is important to note that with the observed flow rate deviations of 10–20%, it would not be possible to implement the feeding system “as is”. However, we see the potential of this unit to be part of a manufacturing process as it overcame the cohesivity and stickiness API attributes (see particle size distribution and ***H***_**R**_ data in Table VII) and was able to dispense the material at very low rates. During the micro-feeding process, the powder is actively transported over all contact surfaces. It is pushed upward *via* the piston on the plate and is moved by the scraper from the plate onto the balance. Therefore, the potential for sticking on free surfaces is small. During all test runs, no significant adhesion or agglomeration was observed and the full amount of powder in the cartridge was collected at the end on the balance (no powder was lost during the feeding process), except for silicon dioxide where small amounts may have lost.

The unit may be able to allow for single API dispensing and allow manufacturers to move away from having to process APIs with excipients to allow for feeding operations (*e.g.*, APIs being mixed with excipients and flow aids to improve their flowability). Thus, the novelty of the micro-feeder system is to overcome a powder’s poor flowability allowing the feeding of very fine and cohesive powders reproducibly, as described by the displacement-feed factor curve. Moreover, by using a combination between the micro-feeder and a loss-in-weight setup, precise and low powder flow rates—required for a commercial scale process—may be achieved.

## CONCLUSIONS

In this study, the feeding performance and the impact of powder properties on feeding were analyzed and discussed for four excipient materials. In addition, one API and one SDI (both highly cohesive) were evaluated to highlight the industrial applicability of the micro-feeder. A density-displacement curve was plotted for all materials investigated. The shape of the curve is highly reproducible, yet different for each material depending on powder properties. We found that large- and small-particle systems had qualitatively the same density-displacement curves; *i.e.*, di-calcium phosphate and CapsuLac60 had similar curves, significantly different from the small-particle system (GrabuLac200 and croscarmellose sodium). For a specific material, these curves are independent from the feed rate. Therefore, these data can be used to calibrate the syringe pump to ensure a constant accurate feed rate. In summary, the following conclusions can be made:

- Low feeding rates down to 1 g/h can be achieved consistently for the tested materials.
- A wide variety of different powders, even low-density cohesive materials, can be fed.
- Repeatability and consistency of feeding, as described by the displacement-feed factor curve, were good. Most importantly, the density-displacement plot is unique for each material. We call this a “displacement-feed factor”. The dependence can be used to control piston speed to achieve a constant and low feed rate for a range of feeding rates (1–20 g/h tested in the study).
- Segregation is not a significant issue in this process (which is another major advantage of the proposed micro-feeding system).

A major benefit of the micro-feeder system is that it requires less material for testing when compared with other feeders (*i.e.*, tens of mL of powder can be used to test micro-feeding system compared with 1–2 kg of powder needed for larger feeders). This is important for highly potent (and expensive) APIs and also during early stages of development.

Future work will address the closed-loop control of this feeder, making it ready for integration in GMP continuous manufacturing lines given the need to better control the flow rate over time. For automation of the system, finger tapping (strokes by the side in the cartridge) during the pre-conditioning process would be replaced by automatically hammering the cartridge periodically during the filling process.
